# Desafios na Realização de Estudos de Evidências do Mundo Real em Países de Renda Média-Baixa: O Estudo EdoBRA

**DOI:** 10.36660/abc.20250353

**Published:** 2025-07-01

**Authors:** Ayrton Roberto Massaro

**Affiliations:** 1 Hospital Sírio-libanês – Neurologia São Paulo SP Brasil Hospital Sírio-libanês – Neurologia, São Paulo, SP – Brasil

**Keywords:** Fibrilação Atrial, Inibidores do Fator Xa, Acidente Vascular Cerebral

Em 1991, Wolf et al. no estudo de Framingham demonstraram que a fibrilação atrial (FA) constitui um fator de risco independente para acidente vascular cerebral (AVC) isquêmico.^1^ Hart et al., em uma metanálise, confirmaram a eficácia da varfarina na redução do risco de AVC em 64% em pacientes com FA.^2^ Observou-se um ligeiro aumento na hemorragia extracraniana grave, o que não impactou a eficácia geral. Farmacocinética imprevisível, interações e necessidades de monitoramento do INR limitaram o uso da varfarina. Em países de baixa e média renda, como o Brasil, as disparidades socioeconômicas afetaram o uso da varfarina em pacientes com FA.^3^

Os anticoagulantes orais diretos (ACODs) foram introduzidos para superar as barreiras associadas à varfarina. Todos os ACODs demonstraram forte prevenção de AVC em comparação com a varfarina. A edoxabana, no estudo ENGAGE AF-TIMI 48, não foi inferior à varfarina em segurança e foi superior na prevenção de AVC hemorrágico.^4^ Pacientes latino-americanos apresentaram taxas de hemorragia e mortalidade mais elevadas em comparação com outras regiões.^5^ Além disso, considerações sobre custo, monitoramento renal, ACODs em subpopulações com FA e agentes de reversão para hemorragia destacam a importância de adaptar a terapia com base nos riscos específicos do paciente.

O século XXI testemunhou um progresso substancial no tratamento do AVC isquêmico agudo por meio de técnicas diagnósticas aprimoradas. A eficácia do tratamento do AVC agudo exigiu centros especializados, beneficiando pacientes em vários estágios da doença, incluindo a prevenção, onde as famílias participam de programas multidisciplinares. O aumento da longevidade global, devido à melhoria dos cuidados de saúde, resultou na sobrevivência de pacientes com condições cardiovasculares diferentes das gerações anteriores, frequentemente com múltiplas comorbidades. Portanto, o risco e a gravidade dos sintomas de FA aumentam com as comorbidades, sugerindo que a prevenção do AVC pode ser mais eficaz se forem abordadas etiologias concorrentes em pacientes com AVC com FA.^6^

A tomada de decisão clínica para FA atualmente depende da classificação binária. No entanto, o desenvolvimento de conceitos para subgrupos de FA pode melhorar a comparabilidade dos resultados em ensaios clínicos. A carga de FA, definida como o tempo gasto em FA durante o monitoramento, é recomendada para avaliação de risco e decisões de tratamento em condições relacionadas à FA, comparando-a com eventos cardiovasculares adversos graves.^7^ Ainda não se sabe se o menor risco de AVC em pacientes com FA pós-AVC justifica diferentes limiares de carga de FA para anticoagulação em comparação à prevenção primária.^8^ Portanto, avaliar a carga de FA em relação à relação risco-benefício é crucial para revisar os achados recentes na epidemiologia de pacientes com FA.^7^

Nesta edição do ABC Cardiol, Précoma et al.^9^ apresentamos o estudo EdoBRA, uma investigação prospectiva conduzida em 30 centros brasileiros que avaliou a edoxabana em pacientes com FA ao longo de 1 ano. De 713 pacientes, 590 concluíram o estudo. A taxa de descontinuação do tratamento foi de 21%, superior à de outros estudos de edoxabana em contexto real, o que pode afetar a interpretação dos desfechos.^10^ O estudo incluiu pacientes em uso de edoxabana por 14 a 90 dias antes do recrutamento, introduzindo viés de seleção ao excluir descontinuações precoces.^9^ Não houve um cronograma específico para avaliação de prontuários com dados ausentes, particularmente para depuração de creatinina.

Eventos adversos foram o principal motivo da descontinuação, com restrições financeiras também contribuindo. Esses achados apresentam limitações, incluindo o impacto da COVID-19. O estudo carece de uma análise de custo-efetividade, o que é particularmente relevante em um país onde fatores socioeconômicos impactam significativamente o acesso à saúde.^9^ A omissão de variáveis críticas pelo estudo limita sua compreensão do impacto socioeconômico na população.

A população do estudo era jovem, predominantemente masculina, não branca e sem plano de saúde, com altas taxas de comorbidades, especialmente insuficiência cardíaca e AVC, refletindo pacientes complexos do mundo real.^9^ A população era predominantemente do Nordeste do Brasil, o que limitou a generalização dos resultados. Esse perfil confirma os achados sobre as características da carga de FA em países com disparidades socioeconômicas, como países de baixa e média renda LMIC (ELSA).^11^ Nessas regiões, quando comparadas a países de alta renda, a doença tende a se manifestar mais precocemente e está associada a uma alta concentração de fatores de risco vascular, que facilitam a ocorrência de comorbidades vasculares adicionais.

A baixa incidência de eventos cardiovasculares e hemorrágicos neste estudo, apesar da população de alto risco, sugere que o edoxaban pode ser particularmente eficaz e seguro para esta coorte, conforme demonstrado anteriormente entre pacientes com latino-americanos no estudo ENGAGE AF-TIMI 48.^5,9^ No entanto, essas descobertas exigem interpretação cautelosa, considerando as implicações de um período de acompanhamento limitado a apenas um ano para uma condição crônica como a FA.
