# Supplementary material for: Desafios na Realização de Estudos de Evidências do Mundo Real em Países de Renda Média-Baixa: O Estudo EdoBRA
Source: Arq Bras Cardiol. 2025 Jul 1;122(4):e20250353. [Article in Portuguese] doi: 10.36660/abc.20250353 (PMC12269928; doi:10.36660/abc.20250353)
Supplement: Translation

# Translation: Challenges in Performing Real-World Evidence Studies in Low-Middle Income Countries: the EdoBRA Study

**DOI:** 10.36660/abc.20250353i

**Authors:** Ayrton Roberto Massaro

**Affiliations:** 1 São Paulo SP Brazil Hospital Sírio-libanês – Neurologia, São Paulo, SP – Brazil

**Keywords:** Atrial Fibrillation, Factor Xa Inhibitors, Stroke

In 1991, Wolf et al. on the Framingham study demonstrated that atrial fibrillation (AF) constitutes an independent risk factor for ischemic stroke.^1^ Hart et al., in a meta-analysis, confirmed warfarin's effectiveness in lowering stroke risk by 64% in AF patients.^2^ A slight increase in major extracranial hemorrhage was observed, which did not impact overall efficacy. Unpredictable pharmacokinetics, interactions, and INR monitoring needs have limited warfarin use. In low-middle income countries (LMICs) like Brazil, socio-economic disparities affected warfarin use in AF patients.^3^

Direct oral anticoagulants (DOACs) were introduced to overcome the barriers associated with warfarin. All DOACs showed strong stroke prevention compared to warfarin. Edoxaban in the ENGAGE AF-TIMI 48 trial was non-inferior to warfarin in safety and superior in preventing hemorrhagic strokes.^4^ Latin American patients showed higher hemorrhage and mortality rates compared to other regions.^5^ In addition, considerations of cost, renal monitoring, DOACs in AF subpopulations, and reversal agents for hemorrhage highlight the importance of tailoring therapy based on patient-specific risks.

The 21^st^ century has seen substantial progress in acute ischemic stroke management through enhanced diagnostic techniques. The efficacy of acute stroke care has necessitated specialized centers, benefiting patients at various disease stages, including prevention, where families join multidisciplinary programs. Increased global longevity from improving healthcare has resulted in patients surviving with cardiovascular conditions different from previous generations, often with multiple comorbidities. Therefore, the risk and severity of AF symptoms rise with comorbidities, suggesting stroke prevention may be more effective if addressing competing stroke etiologies in AF-stroke patients.^6^

Clinical decision-making for AF currently relies on binary classification. However, developing concepts for AF subgroups could improve outcome comparability in trials. AF burden, defined as time spent in AF during monitoring, is recommended for risk evaluation and treatment decisions in AF-related conditions, comparing it with major adverse cardiovascular events.^7^ It remains uncertain if lower stroke risk in post-stroke AF patients justifies different AF burden thresholds for anticoagulation compared to primary prevention.^8^ Therefore, evaluating the AF burden against the benefit-risk ratio is crucial for reviewing recent findings in the epidemiology of AF patients.^7^

In this issue of the ABC Cardiol, Précoma et al.^9^ present the EdoBRA study, a prospective investigation conducted across 30 Brazilian centers that assessed edoxaban in AF patients over 1 year. Of 713 patients, 590 completed the study. The treatment discontinuation rate was 21%, higher than in other real-world edoxaban studies, which may affect outcome interpretation.^10^ The study included patients on edoxaban for 14 to 90 days before enrollment, introducing selection bias by excluding early discontinuations.^9^ There was no specific schedule for evaluating records with missing data, particularly for creatinine clearance.

Adverse events were the main reason for discontinuation, with financial constraints also contributing. These findings have limitations, including the impact of COVID-19. The study lacks a cost-effectiveness analysis, which is particularly relevant in a country where socio-economic factors significantly impact healthcare access.^9^ The study's omission of critical variables limits its understanding of the socio-economic impact on the population.

The study population was young, predominantly male, non-white, and uninsured, with high comorbidity rates, especially heart failure and stroke, reflecting complex real-world patients.^9^ The population was mainly from Northeast Brazil, which limited the generalizability of the results. This profile confirms findings on AF burden characteristics in nations with socio-economic disparities, such as LMIC (ELSA).^11^ In these regions, when compared with high-income countries, the disease tends to manifest earlier and is associated with a high concentration of vascular risk factors, which facilitate the occurrence of additional vascular comorbidities.

The low incidence of cardiovascular and bleeding events in this study, despite the high-risk population, suggests that edoxaban may be particularly effective and safe for this cohort, as demonstrated previously among latin american patients in the ENGAGE AF-TIMI 48 trial.^5,9^ However, these findings require cautious interpretation, considering the implications of a follow-up period limited to only one year for a chronic condition such as AF.
